# Adapting practices to accelerate the scientific description of invertebrate cryptic species

**DOI:** 10.1098/rsbl.2025.0385

**Published:** 2025-10-01

**Authors:** Régis Vivien, Patrick Martin, Jan Pawlowski, Roman Alther

**Affiliations:** ^1^ Swiss Centre for Applied Ecotoxicology, Lausanne/Dübendorf, Switzerland; ^2^ Royal Belgian Institute of Natural Sciences, Brussels, Belgium; ^3^ Institute of Oceanology, Polish Academy of Sciences, Sopot, Poland; ^4^ Department of Aquatic Ecology, Swiss Federal Institute of Aquatic Science and Technology (Eawag), Dübendorf, Switzerland; ^5^ Department of Evolutionary Biology and Environmental Studies, University of Zurich, Zurich, Switzerland

**Keywords:** cryptic species, formal species description, practices for species description, invertebrates

## Abstract

Formally describing cryptic species is essential for conservation and protection purposes and to enable their use in environmental monitoring. The current scientific practice in many invertebrate groups requires assigning the original morphospecies name to a particular genetic lineage before formally describing the other lineages of the morphospecies and providing an exhaustive morphological characterization for each described lineage of the morphospecies. These practices considerably delay—and may even hinder—the scientific description of cryptic species. Furthermore, it may lead to confusion if the same name refers to both the entire morphospecies and a particular lineage. Here, we propose some recommendations to accelerate the description of cryptic species and avoid taxonomic confusion. They include assigning a new name to each lineage of the morphospecies without (necessarily) first obtaining DNA from the morphospecies holotype or paratype(s) or designating a neotype, providing a basic morphological diagnosis in the cryptic species descriptions, and systematically following the morphospecies names by ‘*sensu lato*’ or ‘species group’ when referring to the entire morphospecies and by ‘*sensu stricto*’ when referring to the original lineage. Our recommendations could contribute to rapidly increasing the proportion of scientifically described cryptic species and enhancing the consideration of cryptic species in ecological assessments and conservation/protection programmes.

## 1. Introduction: the importance of describing cryptic species

Biological speciation can occur without any visible morphological modifications [1], resulting in cryptic diversity. It is widely recognized that cryptic species are common among metazoans, forming morphospecies (i.e. cryptic species complexes). Numerous cryptic species have been identified in recent years within common morphospecies, among others in such groups as oligochaetes (e.g. *Tubifex tubifex*, *Limnodrilus hoffmeisteri*), amphipods (e.g. *Gammarus fossarum*), insects (e.g. *Encyrtus sasakii*) or in helminth parasites of human and veterinary importance [2–7]. These cryptic species should be treated as distinct species [8,9], yet, to our knowledge, only a small fraction of them have been formally described.

The criteria used to determine whether different (genetic) lineages within a morphospecies represent distinct species should ideally be integrative—that is, based on all available data from the literature, including morphology, molecular sequences, behaviour, ecology and reproductive isolation [1,10]. However, only certain characteristics may be relevant for distinguishing cryptic species within a particular group [11,12]. For example, behavioural and reproductive isolation experiments can be carried out on some species, such as terrestrial oligochaetes [13–15] but are not feasible for rare species or those that are difficult or nearly impossible to isolate and cultivate. For most cryptic metazoan species, DNA sequences are likely the only effective—yet sufficient—tool for delimitating and describing species (e.g. [16–18]). The International Code of Zoological Nomenclature (ICZN) does not require that the descriptions of species are based solely on morphological characteristics [11].

Referring to cryptic species as operational taxonomic units (OTUs), molecular OTUs (MOTUs), putative species, genetic types, etc., effectively denies their existence as real species and results in their omission from species inventories [11]. Using informal names across studies impedes the comparison of results between studies and in consequence makes global biodiversity analyses difficult. Once their validity is established—based on DNA data and, where possible, supported by additional evidence—the cryptic species should be formally described as nominal species and recognized by the scientific community as integral components of biodiversity [4,18].

Scientifically describing cryptic species is essential for conservation and protection purposes, but also because cryptic species within a morphospecies may differ in ecological, ecotoxicological [19,20] and physiological traits. For instance, cryptic species may exhibit varying degrees of resistance to specific contaminants or contaminant groups [2]. Considering individual cryptic species—rather than morphospecies as a whole—in ecological studies could significantly enhance the accuracy of ecological assessments [21]. The recognition of cryptic species as nominal species would also facilitate the acquisition of knowledge on their ecology, as it would make the comparison of their ecology between the different studies easier. Therefore, cryptic species should be formally described as soon as they are detected.

In studies based on (e)DNA metabarcoding (e.g. [22]) or high-throughput barcoding (i.e. NGS barcoding) [23,24], sequences of cryptic species within a morphospecies that are not described as distinct species are taxonomically assigned to the morphospecies level, thus to a single nominal ‘species’. Accounting for these undescribed cryptic species requires re-analysing metabarcoding data by constructing barcode or phylogenetic trees and by applying methods for lineage delimitation and species discrimination. This process is methodologically complex and time-consuming, posing a major obstacle to the routine recognition of cryptic species in large-scale biodiversity studies.

To allow cryptic species to be described shortly after their detection, an adaptation of the current practices in the description of cryptic species is required. The aim of this article is to identify the issues posed by these practices and to address them by proposing some recommendations that are fully compliant with ICZN rules.

### 2. Current practices in cryptic species description and drawbacks of such practices

**Figure 1.**
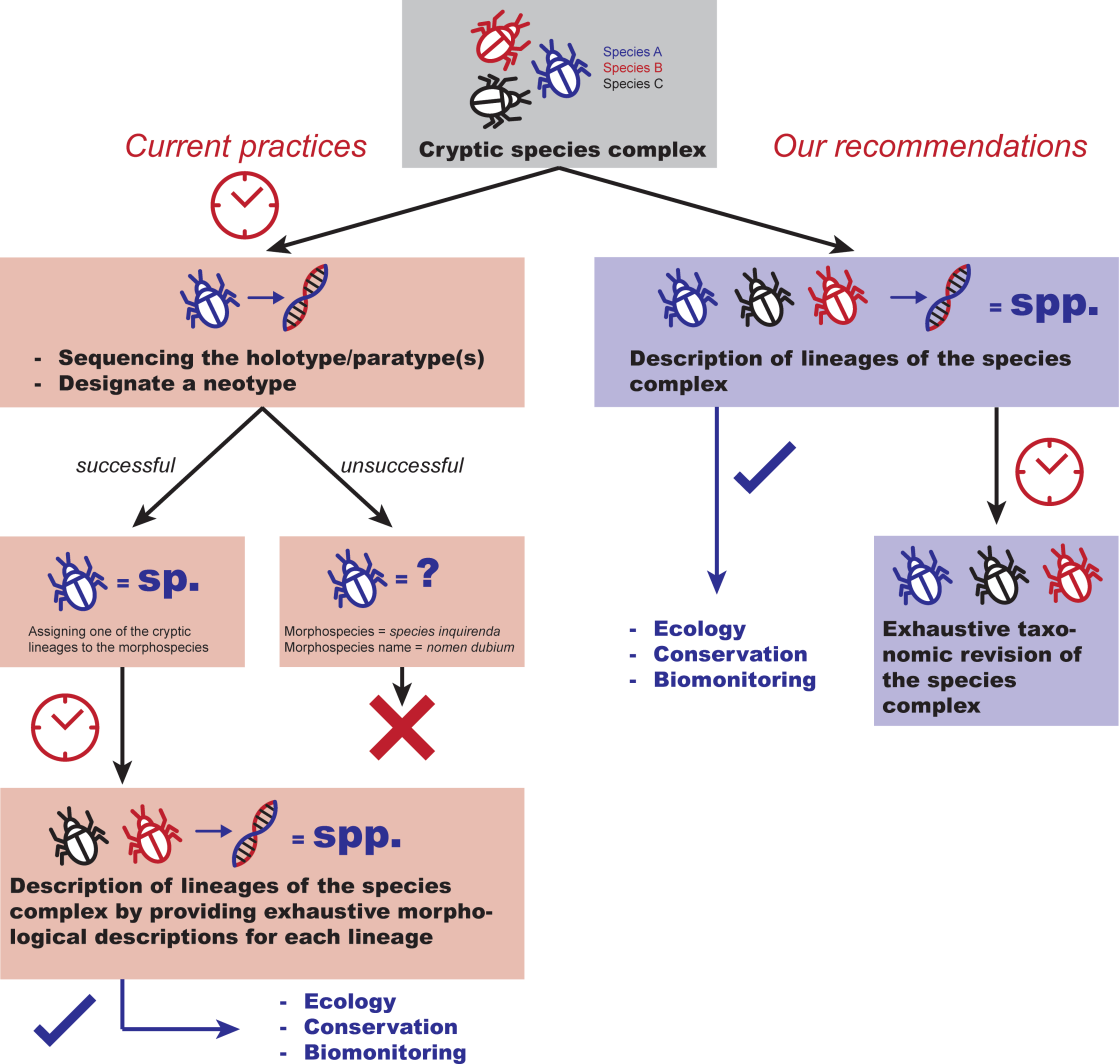
Diagram comparing the current practices and the modified practices suggested in the present paper for formally describing cryptic species.

Currently, in specific groups, such as oligochaetes and amphipods, but also in many other groups of invertebrates, it is generally accepted that one of the cryptic species (i.e. a genetic lineage) within a morphospecies should retain the original morphospecies name—that is, the name assigned by the first descriptor of the morphospecies—while each of the remaining lineages should be given a new species name [25] (figure 1; electronic supplementary material, figure S1). In many cases, especially for species described in the nineteenth or early twentieth centuries, the holotype and paratype(s) have been lost, destroyed, never preserved or preserved in ways that render them unsuitable for molecular analysis (e.g. mounted between slide and coverslip, or fixed in low-pH formalin). In such cases, the ICZN allows for the designation of a neotype under specific conditions (Art. 75.3), particularly the requirement in Article 75.3.6 that the neotype originates from the same locality as the original type material, or as close as possible [18]. This makes it theoretically possible to assign the original morphospecies name to a genetic lineage by collecting new material from or near the type locality.

This approach presents numerous practical and taxonomic challenges. Attributing the morphospecies name to a lineage through sequencing of historical material or neotype designation requires locating and accessing the original type locality or museum material, performing genetic analyses on often poorly preserved specimens and collecting fresh material from potentially distant and hardly accessible locations. These challenges can discourage researchers from formally describing cryptic species.

Designating a neotype (with a high degree of confidence) poses different problems and can even prove impossible. First, the original description of a morphospecies may have been based on the examination of specimens belonging to two or more lineages if several lineages of the morphospecies coexist at the same locality—a common situation for example in the groups *T. tubifex*, *L. hoffmeisteri* [23], *Chaetogaster diastrophus* [26] and *Niphargus podpecanus* [27]. Second, it is often uncertain whether a newly designated neotype corresponds to the same lineage as the original holotype. Destruction or modification of the habitat at type locality (e.g. urbanization), the presence of multiple co-occurring lineages and vague type localities further increase the risk of incorrect neotype designation. This could lead to significant taxonomic complications if the neotype is later shown to represent a different lineage. Third, information on the type locality is often missing [25]—as exemplified by *C. diastrophus* [28], whose type locality is not documented [26]. Fourth, collecting specimens of the sought morphospecies at the type locality or close to it could prove challenging or unsuccessful if the abundance of the morphospecies is low.

Researchers often delay the description of cryptic species until they can obtain DNA from the morphospecies holotype/paratype(s) or designate a neotype, in order to avoid assigning a new name to the lineage corresponding to the holotype [29]. Despite significant effort, they often fail retrieving or sequencing the morphospecies holotype/paratype(s) and end up designating as neotype a lineage of the morphospecies that could possibly not correspond to the lineage of the holotype.

Another barrier to the formal description of cryptic species is the expectation that each lineage be described morphologically exhaustively, typically involving, for most species, mature specimens examined by dissection. This requirement, although not formally imposed by the ICZN, adds an additional burden and has certainly contributed to the widespread neglect of cryptic species in taxonomy. This burden is further reinforced by the erosion of global morphological taxonomic expertise, a discipline once central to species description prior to the rise of molecular approaches [30–32]. Finding distinctive morphological features between cryptic species, that are, by essence, identical or very similar could prove challenging or impossible, even if the work is performed by a skilled taxonomist. Such thorough examination may lead to inconclusive results, as illustrated by the study on the cryptic species of *Branchiodrilus* (Clitellata, Naididae) [33]. The genus *Haplotaxis* (Clitellata, Haplotaxidae) provides another example. Despite his expertise, Michaelsen [34], then regarded—and still recognized—as one of the foremost authorities on oligochaetes [35], was unable to distinguish the European and North American species described up to that time and placed them all in synonymy. This taxonomic situation remained largely unchanged until recently but has now been challenged by a genetic study of *Haplotaxis gordioides* conducted in Switzerland [29].

Finally, further complications arise from the dual use of the morphospecies name to refer both to the entire morphospecies complex (based on morphological identification) and to a particular genetic lineage of the morphospecies, often without clarification. This ambiguity leads to confusion in public DNA reference databases, where users are unable to distinguish between sequences corresponding to the morphospecies as a whole—and potentially corresponding to different lineages of the morphospecies—and those corresponding to a particular lineage.

## 3. Recommendations

We here propose some recommendations to address the issues posed by the current practices in dealing with cryptic species (figure 1; electronic supplementary material, figure S1).

First, we recommend assigning a new name to each lineage of the morphospecies without the prevalent expectation of first attempting to obtain DNA from the morphospecies holotype/paratype(s) or to designate a neotype. We emphasize that this suggestion applies when the morphospecies holotype/paratype(s) are not easily accessible and/or may be unsuitable for molecular analyses (e.g. if they are very old) or when a neotype cannot be readily designated.

Second, we recommend providing morphological descriptions of cryptic species allowing for identification at the morphospecies level, and not necessarily an exhaustive morphological descriptions, so as not to delay the description of cryptic species. A comprehensive morphological description of each lineage (for example, based on the morphology of extracted genital organs), which requires very specific and rare expertise and examination of specimens in a certain development stage, does not need to be included and could be performed at a later time. In any case, such an extensive description would likely conclude, as mentioned previously, that there are no or only minor—and possibly ambiguous/doubtful—morphological differences between the cryptic species of a given morphospecies.

Third, we recommend that the description of cryptic lineages includes, at minimum and among other elements: a reference to the morphospecies, the geographical coordinates of the sampling locality, a morphological description (at the morphospecies level) of at least one specimen from the lineage (with eventually some drawings), some photos of the morphological features used for the identification, information on the ecology of the lineage (when possible), the reference numbers of the holotype and paratype(s) deposited in a museum or another institution, the sequences of selected genetic markers, in accordance with current standards for species delimitations (e.g. [9,18,27,29]) and a molecular diagnosis (e.g. the approach Character Attribute Organization System). The latter is a set of characters (i.e. specific positions within the sequence) and character states (i.e. the nucleotide present at each of those positions) whose presence in an individual supports its assignment to the species in question [27].

Finally, we recommend systematically indicating ‘species group’ or ‘*sensu lato*’ after the morphospecies name when referring to the morphospecies level (i.e. morphological identifications), as proposed by Schmelz *et al.* [25]. If, after assigning a new name to each lineage within a morphospecies, one of these lineages can be unanimously attributed to the morphospecies holotype—either through direct sequencing of the morphospecies holotype/paratype(s) or by designating a neotype with a high degree of confidence (e.g. specimens collected at the type locality, presence of only one cryptic species at the type locality)—the new name of that lineage would be replaced with the original morphospecies name, rendering the new name a junior synonym. To avoid the taxonomic confusion mentioned above, the morphospecies name should be systematically followed by ‘*sensu stricto*’ when it refers to the original lineage. As long as the morphospecies cannot be confidently assigned to a lineage, it should be provisionally considered as *species inquirenda*, meaning that the taxonomic identity of the species remains unclear. If it proves impossible to resolve this uncertainty (e.g. due to the loss of type material and unknown type locality), the name of the morphospecies should be treated as *nomen dubium*, as suggested by Schmelz *et al.* [25].

## 4. Discussion and conclusion

The proposed adaptations to current practices for describing cryptic species—consisting of assigning a new species name to each lineage of the morphospecies without the expectation of first attempting to obtain DNA from the morphospecies holotype/paratype(s) or to designate a neotype and of providing morphological descriptions that capture features common to all molecular lineages of the morphospecies rather than comprehensive morphological descriptions—would accelerate the process of scientific description of cryptic species. Attributing a lineage to the holotype of the morphospecies and providing exhaustive morphological description of the lineages to detect eventual subtle differences between them would still be possible later, without delaying or hindering the description of cryptic species within the morphospecies. Moreover, systematically using the terms ‘*sensu lato’* or ‘species group’ in public reference databases when referring to the entire morphospecies, and ‘*sensu stricto*’ when referring to a particular lineage, would enable taxonomic confusion to be avoided.

It has been suggested that, when the holotype/paratype(s) of the morphospecies cannot be retrieved or sequenced and a neotype cannot be designated with a high degree of confidence (e.g. if no specimen of the targeted morphospecies can be sampled at the type locality, or if the type locality is unknown), any lineage of the morphospecies may be arbitrarily attributed to the morphospecies name in order to stabilize the nomenclature (pers. comm.). However, such a practice would not comply with ICZN rules as the requirement of Article 75.3.6 would not be met. Indeed, the probability that the holotype and neotype belong to different lineages would be high. This could result in an incorrect attribution of a lineage to the holotype of the morphospecies, and consequently, the newly attributed lineage may not fully correspond to the original description of the holotype or paratype(s) in terms of morphology and/or ecology. Moreover, an arbitrary neotype designation could lead to incorrect biogeographic and ecological information on the neotype lineage as it may be absent from, or never have been present at, the type locality. Finally, an arbitrary neotype designation could be contested, potentially generating conflicts, and researchers could be dissuaded from designating a neotype with a high degree of confidence if an arbitrary neotype designation were considered acceptable anyway. We advocate either for a unanimous designation of a neotype (high probability that the holotype and neotype belong to the same lineage) or for considering the morphospecies as *species inquirenda*, or its name as a *nomen dubium*.

Informal description of cryptic species by attributing them codes, such as numbered MOTUs, OTUs or other types, has important drawbacks. First, this practice does not ensure that lineages are constantly designated with the same codes across studies nor that comprehensive molecular investigations were conducted to determine whether lineages with the same species name and different codes represent distinct species (rather than just phylotypes). The formal description of cryptic species ensures that they are named consistently across studies and are legally recognized as distinct species.

We emphasize that, in our approach, formal descriptions based solely on molecular data are not encouraged. Instead, we recommend providing the most detailed morphological descriptions possible, ideally including an exhaustive examination whenever the expertise is available and when the holotype and paratype(s) are in suitable condition (e.g. fully mature). Moreover, to reliably link cryptic lineages to morphospecies, we consider that a lineage should be formally described only when the diagnostic morphological features of the morphospecies are visible in the holotype and paratype specimens. Accordingly, we recommend systematically providing at least the morphological characters necessary for identification at the morphospecies level. This practice also ensures that each description is self-contained and does not depend on reference to the original morphospecies description.

We acknowledge that this proposal has some drawbacks. First, it carries the risk of increasing the number of junior synonyms. Second, this approach tends to defer the labour-intensive task of morphological description—not only to a later stage, but more problematically, to other researchers. If taxonomists are left to describe species that have already been named, and thus cannot be credited with their nomenclature, it is uncertain whether sufficient motivation will remain to undertake such work. To mitigate this issue, it is essential to involve morphological taxonomists directly in the naming process, even when species are diagnosed exclusively by molecular data. Doing so would afford them the necessary time to investigate subtle morphological characters, where present, and to supplement molecular diagnoses with rigorous morphological descriptions.

Nevertheless, we argue that given the urgent need to formally describe cryptic species, our proposal provides significant advantages that largely outweigh its potential drawbacks. Moreover, these disadvantages should be weighed against the far more consequential drawbacks of current practices, which lead to delay or hinder the description of cryptic species. By facilitating the process and removing obstacles, this approach could contribute to rapidly increase the proportion of scientifically described cryptic species and enhance their integration into ecological assessments and conservation/protection programmes. When developing this approach, we ensured full compliance with ICZN rules, allowing for broad acceptance by the scientific community. We, therefore, emphasize that this approach can be immediately applied across all invertebrate groups. Our recommendations are intended to leverage novel techniques and provide the tools necessary to address the growing global biodiversity crisis.

## Data Availability

This article has no additional data. Supplementary material is available online [36].
